# Biochemical and structural characterization of a recombinant fibrinogen-related lectin from *Penaeus monodon*

**DOI:** 10.1038/s41598-021-82301-5

**Published:** 2021-02-03

**Authors:** Nongnuch Singrang, Sirasit Laophetsakunchai, Bich Ngoc Tran, Paul T. Matsudaira, Anchalee Tassanakajon, Kittikhun Wangkanont

**Affiliations:** 1 Center of Excellence for Molecular Biology and Genomics of Shrimp, Department of Biochemistry, Faculty of Science, Chulalongkorn University, Bangkok, Thailand; 2 Molecular Crop Research Unit, Department of Biochemistry, Faculty of Science, Chulalongkorn University, Bangkok, Thailand; 3 Department of Biological Sciences, Faculty of Science, Centre for BioImaging Sciences, National University of Singapore, Singapore, Singapore

**Keywords:** Innate immunity, Glycobiology, Proteins, Structural biology

## Abstract

Fibrinogen-related lectins are carbohydrate-binding proteins of the innate immune system that recognize glycan structures on microbial surfaces. These innate immune lectins are crucial for invertebrates as they do not rely on adaptive immunity for pathogen clearance. Here, we characterize a recombinant fibrinogen-related lectin *Pm*FREP from the black tiger shrimp *Penaeus monodon* expressed in the *Trichoplusia ni* insect cell. Electron microscopy and cross-linking experiments revealed that *Pm*FREP is a disulfide-linked dimer of pentamers distinct from other fibrinogen-related lectins. The full-length protein binds N-acetyl sugars in a Ca^2+^ ion-independent manner. *Pm*FREP recognized and agglutinated *Pseudomonas aeruginosa*. Weak binding was detected with other bacteria, including *Vibrio parahaemolyticus*, but no agglutination activity was observed. The biologically active *Pm*FREP will not only be a crucial tool to elucidate the innate immune signaling in *P. monodon* and other economically important species, but will also aid in detection and prevention of shrimp bacterial infectious diseases.

## Introduction

The black tiger shrimp *Penaeus monodon* is an important economic animal of many countries, including Thailand^1^. However, there are many infectious diseases devastating shrimp farming. Viral pathogen such as white spot syndrome virus^2^, yellow head virus^3^, or shrimp baculovirus^4^ have costed severe economic loss and their molecular properties have been extensively investigated. There are various test kits available and factors in controlling infections are known^5^. Recently, bacterial pathogen such as *Vibrio parahaemolyticus*, causing the acute hepatopancreatic necrosis disease (AHPND), has emerged as a major disruption in the shrimp farming industry^6^. Very little is known about the interplay of the shrimp innate immune system and pathogenic bacteria. One key player is likely the lectins of the innate immune system^7,8^.

Lectins, or carbohydrate binding proteins, play important roles in pathogen recognition, especially in invertebrates where adaptive immunity is not as developed compared to vertebrates^9^. Lectins of the innate immune system generally can distinguish self from non-self by recognition of carbohydrate residues or specific glycan structure not present on the host cells. There are several families of animal lectins that are involved in the innate immunity, such as C-type lectins^10^, ficolins^11^, and intelectins^12,13^ (X-type lectin). Ficolins and intelectins are classified as fibrinogen-like lectins based on structural homology. In mammalian systems, ficolins are known to activate the lectin complement system for pathogen clearance^7^. Ficolins may act as an opsonin for phagocytosis as well^14,15^. However, the sequence of signaling events after bacteria recognition by lectins in invertebrate is largely unknown. Thus, we are interested in investigating the structure and function of fibrinogen-related lectin in shrimp, as it may have applications in bacterial disease prevention and treatment.

**Figure 1:**
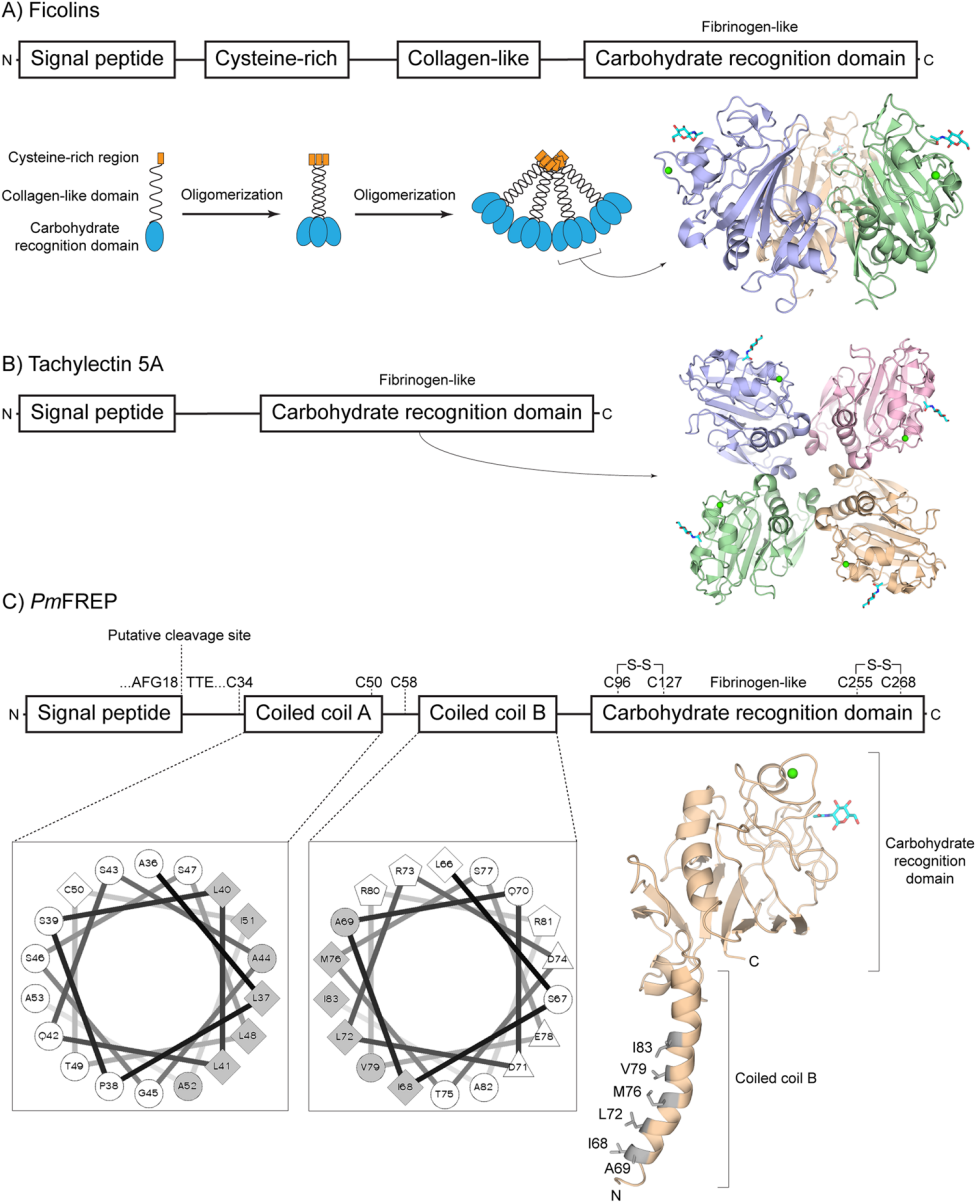
Comparison of fibrinogen-like lectin structures. (**A**) Ficolins. Each polypeptide chain can trimerize and oligomerize into a flower bouquet-like structure. The structure represents a trimeric CRD. (**B**) Tachylectin 5A. The structure represents the tetrameric CRD. and (**C**) *Pm*FREP. The coiled coil regions are also represented with helical wheel diagrams showing the predicted amphipathic helices. The structure shown is a homology model of the CRD and coiled coil B built with human fibrinogen (PDB ID 2HPC) as the template.

Fibrinogen-related lectins are widely distributed in the animal kingdom and they have diverse molecular structure (Fig. 1). Ficolins, found in both vertebrates and invertebrates, contain a cysteine-rich N-terminal region, a collagen-like region, and the carbohydrate recognition domain (CRD) at the C terminus (Fig. 1A). Because of the collagen-like region that is capable of forming a triple helix, ficolins can trimerize. The cysteine-rich region then mediates disulfide-linked oligomerization of the trimer into higher order oligomers, resulting in a fan- or flower bouquet-shaped molecular complex^16,17^. Ficolins binds N-acetyl glucosamine (GlcNAc)-containing glycan and subsequent activate the innate immune system, such as the lectin complement pathway in mammals^18,19^. However, the signaling event in invertebrate is not well-studied. Another group of invertebrate fibrinogen-like lectin is tachylectin 5A (Fig. 1B). The protein has a simple primary structure of only the CRD. The protein is tetrameric and binds GlcNAc^20,21^. The protein is proposed to be involved in bacteria sensing and hemolymph clotting to seal the bacteria-exposed wound, but the molecular mechanism following ligand recognition remains unknown^22^. Recently, a unique fibrinogen-like lectin from *P. monodon* was described. The protein has been cloned by various investigators, and named *Pm*FREP^23^ or PL5-1^24^. *Pm*FREP binds bacterial peptidoglycan^23^, thus likely to bind GlcNAc as observed with ficolins and tachylectin 5A. *Pm*FREP contains three cysteines outside of the CRD (Fig. 1C). Because of the odd number of cysteines, they are likely to participate in intermolecular disulfide bond formation. In contrast to ficolins, *Pm*FREP does not contain the collagen-like sequence, but contains two coiled coil regions that likely for amphipathic helices and mediate higher order oligomerization. Thus, we predict that *Pm*FREP has a novel molecular assembly unique among fibrinogen-like lectins.

*Pm*FREP and related proteins have been purified from *P. monodon* hemolymph^23,24^. Because of the potential contamination of other GlcNAc-binding fibrinogen-related lectins, it is difficult to study the structure and function of *Pm*FREP using the protein purified from the hemolymph. In addition, the complex architecture of *Pm*FREP may cause difficulties in recombinant protein expression. To date investigators that have cloned and studied *Pm*FREP have reported recombinant expression of *Pm*FREP-related proteins, such as PL5-2, in *Escherichia coli*, but not *Pm*FREP (PL5-1) itself^24^. Given the potential intermolecular disulfide bonds and higher-order oligomerization, it is unlikely that *E. coli* and in vitro refolding of inclusion bodies can produce functional *Pm*FREP, or any *Pm*FREP-related proteins. The problem of protein quality is a bottleneck in investigating lectin structure, function, and signaling in *P. monodon*. Therefore, we aim to produce functional recombinant *Pm*FREP to investigate its structure and function.

In this study, we produced recombinant *Pm*FREP for biochemical and structural characterization. Due to the complex architectures of fibrinogen-related lectins, high level of protein expression was successful in insect cells. The dimer of pentamer structure of *Pm*FREP was revealed by both biochemical methods and electron microscopy. Binding to N-acetyl sugars and bacteria agglutination were also demonstrated. These results will not only be crucial for further investigation of immune signaling in shrimp, but will also help in combating bacterial infectious diseases in shrimp.

## Results

*Pm*FREP was cloned into various expression vectors for expression in bacteria (*Escherichia coli*), mammalian cells (HEK293T), and insect cells (*Trichoplusia ni*). In the *E. coli* system, no signal peptide was included in the coding sequences and both the N- and C-terminal hexahistidine (His_6_) tag was explored. Despite exploration of host strains and IPTG concentrations, no protein expression was detected both by SDS-PAGE and western blot against the His_6_ tag (data not shown). Thus, protein expression and secretion were further explored in mammalian and insect cells (Figure S1). Protein expression was barely observable for *Pm*FREP with its native signal peptide and a C-terminal His_6_ tag (Native SP *Pm*FREP His_6_) when expressed and secreted from insect cells. Another expression construct examined is *Pm*FREP with the *Xenopus laevis* embryonic epidermal lectin signal peptide and an N-terminal His_6_ tag (XEEL SP His_6_ *Pm*FREP, Figure S2A). This expression construct yielded higher amount of protein in the insect system compared to the mammalian system. Truncation of *Pm*FREP to only the CRD with the XEEL signal peptide and a N-terminal His_6_ tag (XEEL SP His_6_ *Pm*FREP, Figure S2B) when expressed in insect cells yielded high amount of protein in insect culture media, visible in SDS-PAGE with Coomassie Blue stain. Therefore, the insect expression system was used to express the constructs XEEL SP His_6_ *Pm*FREP and XEEL SP His_6_ *Pm*FREP CRD, yielding His_6_ *Pm*FREP and His_6_ *Pm*FREP CRD respectively. The protein was purified by Ni–NTA affinity chromatography for subsequent experiments (Figure S3). Correct cleavage of the signal peptide for both His_6_ *Pm*FREP and His_6_ *Pm*FREP CRD was verified with N-terminal protein sequencing (Figure S4).

**Figure 2:**
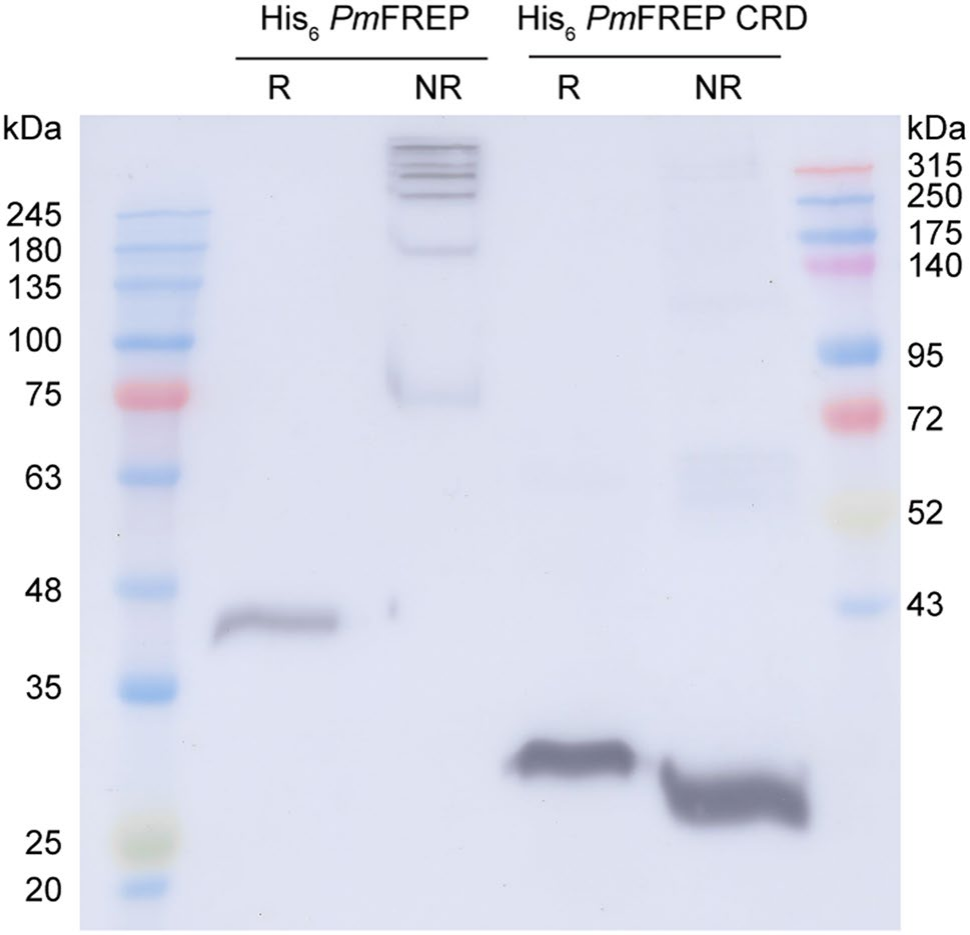
Western blot of His_6_ *Pm*FREP and His6 *Pm*FREP CRD under reducing (R) and non-reducing (NR) conditions. Anti-His_6_ antibody was used as the primary antibody.

With the ability to produce recombinant *Pm*FREP, we next explored the disulfide linked oligomeric states of *Pm*FREP by examining the apparent molecular weight under reducing (with DTT in sample buffer) and non-reducing conditions (no DTT in sample buffer) (Fig. 2). His_6_ *Pm*FREP ran as a single species under reducing conditions, but appear to assemble into large disulfide-linked oligomers of more than 315 kDa. On the other hand, His_6_ *Pm*FREP CRD appeared monomeric both in reducing and non-reducing conditions, indicating that there are no intermolecular disulfide bonds in the CRD.

**Figure 3:**
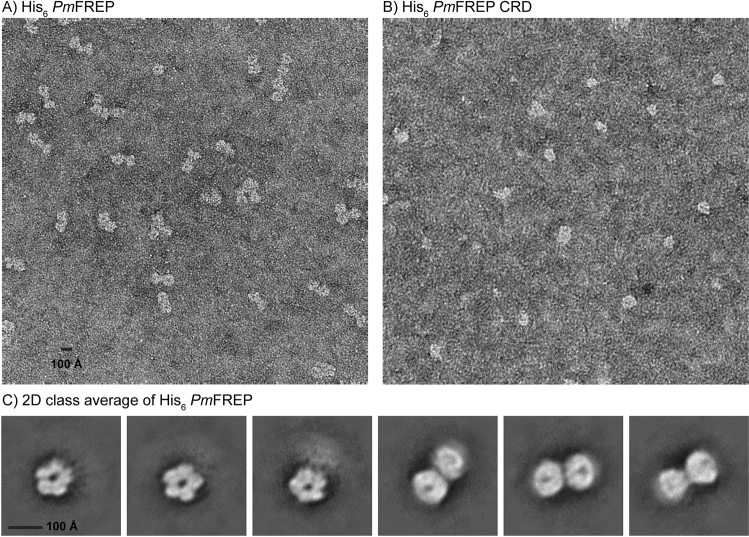
TEM micrograph of (**A**) His_6_ *Pm*FREP, (**B**) His6 *Pm*FREP CRD, and (**C**) 2D class averages of His_6_ *Pm*FREP.

**Figure 4:**
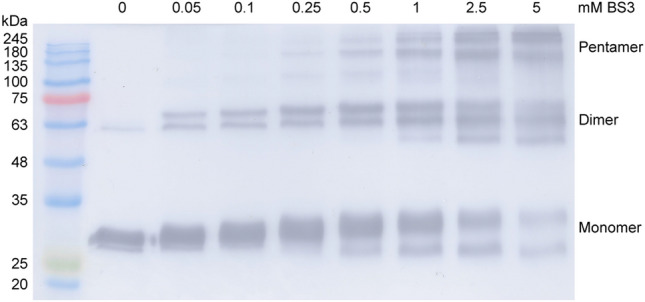
Crosslinking of *Pm*FREP CRD with bis(sulfosuccinimidyl)suberate (BS3) at various concentrations. *Pm*FREP was detected by western blot probed with an anti-His_6_ antibody.

To further examine the quaternary structure of *Pm*FREP, both His_6_ *Pm*FREP and His_6_ *Pm*FREP CRD were examined by transmission electron microscopy (TEM) with negative staining (Fig. 3). His_6_ *Pm*FREP appeared as dumbbell-shaped molecules with the dimension of roughly 100 × 200 Å. Two-dimensional class averaging suggested that the molecule is a dimer of pentamer with a flexible linker in between. This oligomeric structure is distinct from other fibrinogen-related lectins, such as mammalian ficolins, which are flower bouquet-shaped consisting of oligomers of trimers, or intelectins which are also trimer or oligomer of trimers. His_6_ *Pm*FREP CRD appeared as 100 Å particles, but we were not able to obtain consistent 2D class average. Dynamic light scattering (DLS) experiments also confirmed the sizes of His_6_ *Pm*FREP (215 ± 36 Å) and His_6_ *Pm*FREP CRD (97 ± 30 Å) (Figure S5). These results suggested that His_6_ *Pm*FREP CRD might still be able to oligomerize in solution, but the oligomeric conformation may not be as stable as the full length His_6_ *Pm*FREP protein. To examine whether His_6_ *Pm*FREP CRD can oligomerize in solution, chemical cross-linking was performed (Fig. 4). As the concentration of the cross-linker was increased, species with the molecular weight consistent with dimers and pentamers were observed. These results suggested that His_6_ *Pm*FREP CRD can self-associate in solution and were consistent with the TEM and DLS results.

**Figure 5:**
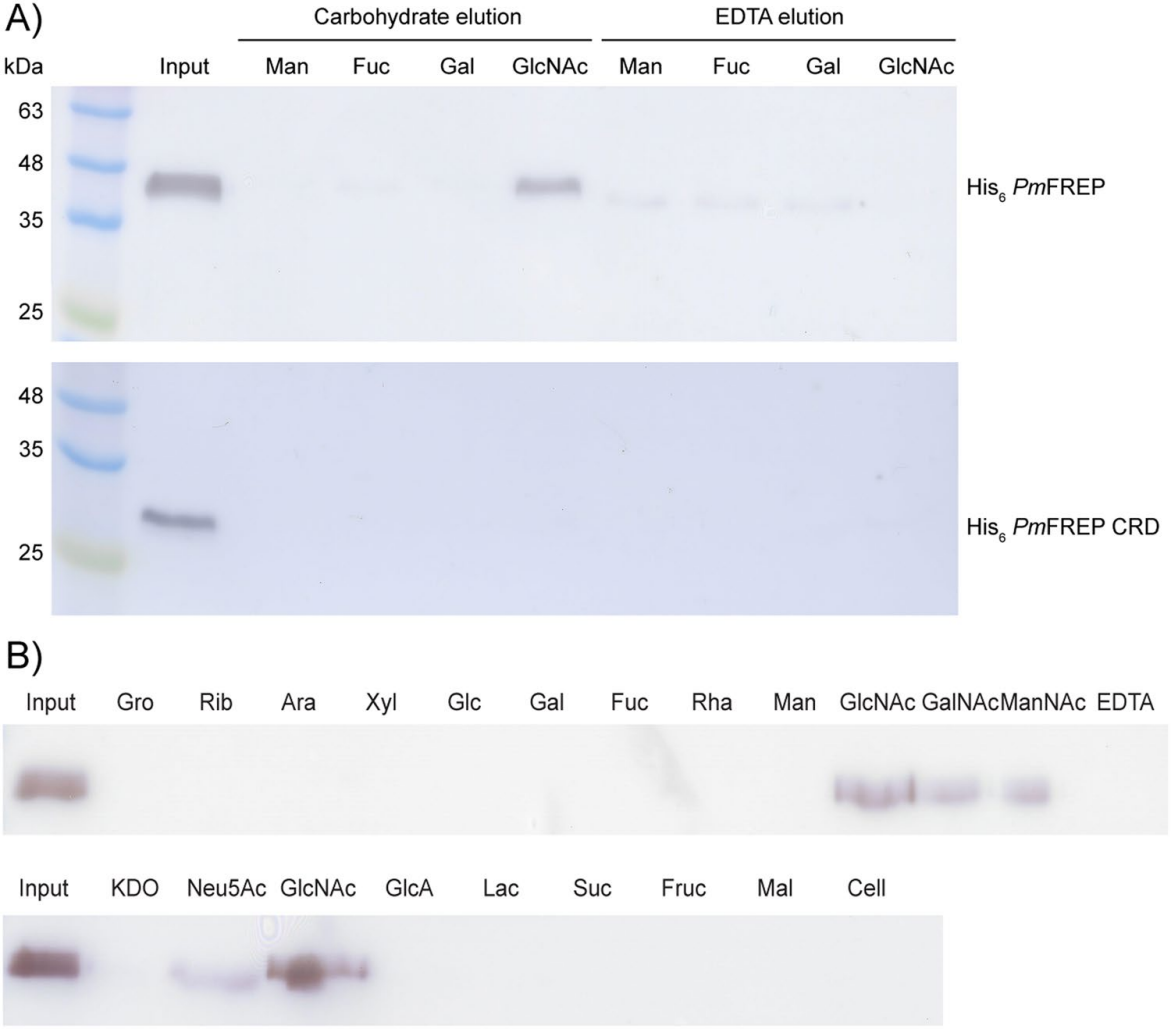
(**A**) Western blot analysis of His_6_ *Pm*FREP (top panel) and His_6_ *Pm*FREP CRD (bottom panel) bound to different carbohydrate resin and eluted with either the respective monosaccharide or EDTA. (**B**) Competitive elution of His_6_ *Pm*FREP bound to GlcNAc resin by different soluble carbohydrates. Anti-His_6_ antibody was used as the primary antibody.

**Figure 6:**
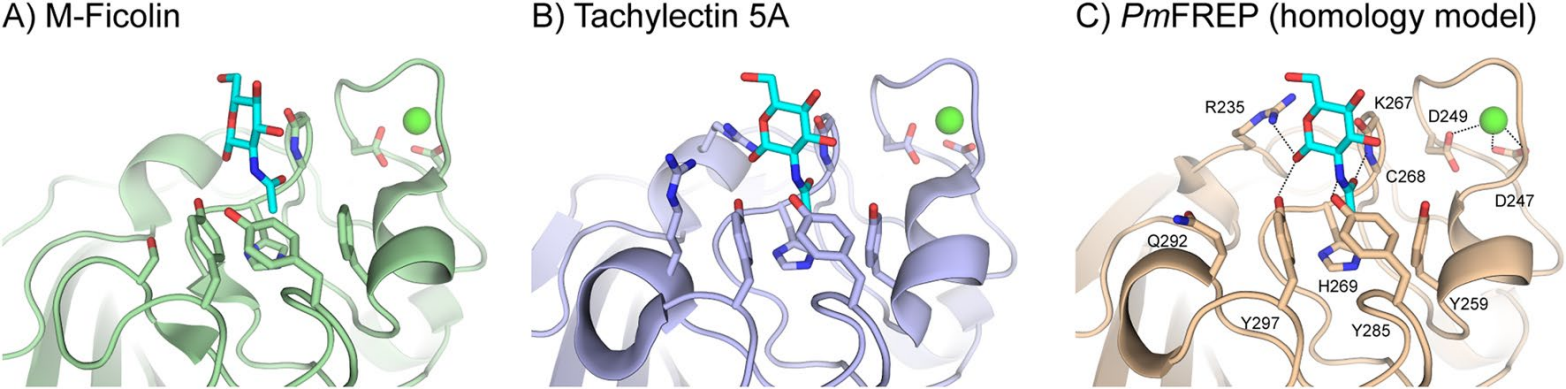
Comparison of the ligand binding site of (**A**) M-ficolin (crystal structure, PDB ID 2JHK), (**B**) Tachylectin 5A (crystal structure, PDB ID 1JC9), and (**C**) *Pm*FREP (homology model using SWISS-MODEL^44^, PDB ID 1JC9 as the template). The GlcNAc ligand is shown in cyan and the structural calcium ion is shown in green.

To explore the ligand binding properties of *Pm*FREP, we performed competitive elution assays (Fig. 5A). The proteins were bound to carbohydrate affinity resins and eluted with the corresponding monosaccharide or EDTA. His_6_ *Pm*FREP can bind GlcNAc as expected for fibrinogen-like lectin. However, EDTA was not able to eluted His_6_ *Pm*FREP from the GlcNAc resin. In contrast to the full length His_6_ *Pm*FREP, His_6_ *Pm*FREP CRD failed to bind any carbohydrate affinity resin. To further explore carbohydrate specificity of His_6_ *Pm*FREP, His_6_ *Pm*FREP was bound to the GlcNAc resin and eluted with various carbohydrates (Fig. 5B). GlcNAc, N-acetylgalactosamine (GalNAc), N-acetylmannosamine (ManNAc), and N-acetylneuraminic acid (Neu5Ac) were able to elute His_6_ *Pm*FREP from the GlcNAc resin. However, competitive elution was not observed with glycerol (Gro), ribose (Rib), arabinose (Ara), xylose (Xyl), glucose (Glc), galactose (Gal), fucose (Fuc), rhamnose (Rha), mannose (Man), 3-deoxy-D-manno-2-octulosonic acid (KDO), glucoronic acid (GlcA), lactose (Lac), sucrose (Suc), fructose (Fruc), maltose (Mal), and cellobiose (Cell). These results suggested that His_6_ *Pm*FREP was specific for acetyl group-containing carbohydrates, and while the ligand binding site and the calcium ion-binding residues were conserved (Fig. 6), the calcium ion is not required for ligand binding.

**Figure 7:**
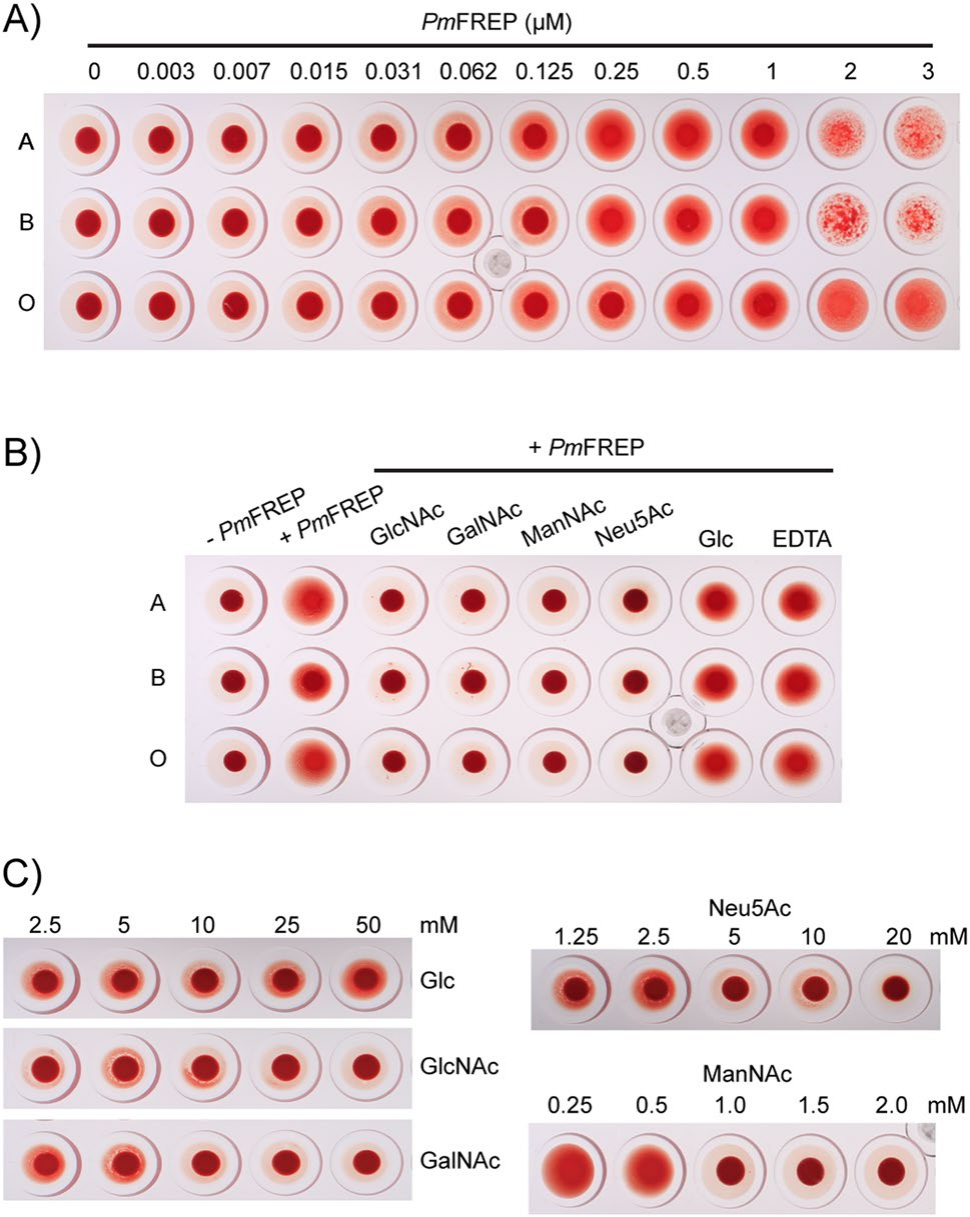
Agglutination of human red blood cells with His_6_ *Pm*FREP. (**A**) Agglutination of different ABO blood type by His_6_ *Pm*FREP at various concentrations. (**B**) Inhibition of ABO blood type agglutination by different carbohydrates (20 mM for Neu5Ac and 50 mM for other carbohydrates) and EDTA. (**C**) Inhibition of A blood type agglutination by different carbohydrates at various concentrations.

To investigate recognition of carbohydrates in cellular context, we examined agglutination of red blood cells by His_6_ *Pm*FREP. Because of the dimer of pentamer arrangement, His_6_ *Pm*FREP is expected to agglutinate cells displaying its ligand. His_6_ *Pm*FREP agglutinates red blood cells of A-, B- and O-type starting at concentration around 0.031 µM (Fig. 7A). Agglutination was inhibited by GlcNAc, GalNAc, ManNAc, and Neu5Ac (Fig. 7B). However, glucose and EDTA could not inhibit agglutination. GlcNAc and GalNAc could inhibit agglutination at around 10 mM (Fig. 7C). Inhibition of agglutination was observed at 5 and 1 mM for Neu5Ac and ManNAc, respectively.

**Figure 8:**
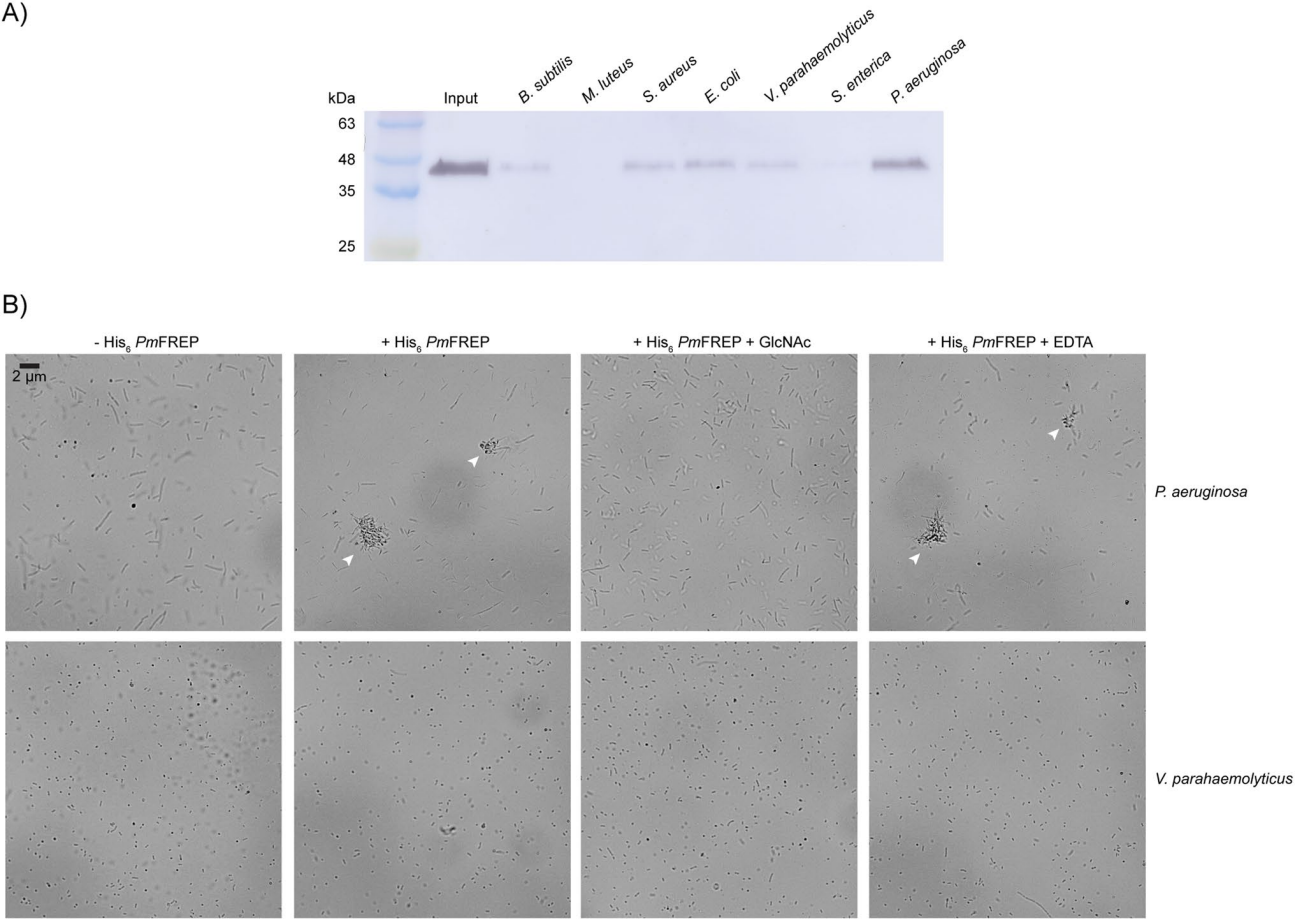
(**A**) Western blot of His_6_ *Pm*FREP eluted from bacteria pellet with 100 mM GlcNAc. Anti-His_6_ antibody was used as the primary antibody. (**B**) Agglutination of bacteria with His_6_ *Pm*FREP (3 µM). Clumps of bacteria are indicated with arrows.

Because of its proposed role in *P. monodon* innate immune system, we next examined the ability of His_6_ *Pm*FREP to recognize bacteria (Fig. 8A). His_6_ *Pm*FREP was bound to bacteria pellet and eluted with GlcNAc. The eluted protein solutions were then examined by western blot. His_6_ *Pm*FREP strongly recognized *Pseudomonas aeruginosa*. Noticeable binding was also observed with *Bacillus subtilis*, *Staphylococcus aureus*, *Escherichia coli*, and *Vibrio parahaemolyticus*. Very little to no binding was observed toward *Micrococcus luteus* and *Salmonella enterica*. Because the dimer of pentamer molecular architecture of *Pm*FREP suggests that the protein can engage two bacteria simultaneously and cause agglutination, bacteria agglutination activity of *Pm*FREP was explored (Fig. 8B). *P. aeruginosa* and *V. parahaemolyticus* were used in the agglutination because of the strong signal in the bacteria binding assay and the importance as a shrimp pathogen respectively. At the highest concentration of His_6_ *Pm*FREP shown to agglutinate red blood cells (3 µM), His_6_ *Pm*FREP agglutinated *S. aureus* and this activity is inhibited by addition of GlcNAc. However, sequestration of Ca^2+^ ion by addition of EDTA did not inhibit agglutination. In contrast, *V. parahaemolyticus* was not agglutinated by His_6_ *Pm*FREP.

## Discussion

Various investigators have purified *Pm*FREP and its homologs from *P. monodon* hemolymph^23–25^. However, the degree of contamination by other homologs is unknown, which complicates further analysis of *Pm*FREP structure and function. Moreover, in contrast to other *Pm*FREP homologs in shrimp, there is no report of expression or characterization of functional recombinant *Pm*FREP^23–25^. We speculate that other investigators have encounter issues with bacterial production of *Pm*FREP. In our hands, we did not observe protein expression in *E. coli* whether the His_6_ tag was placed at the N- or C-terminus. Thus, we conclude that bacterial expression system might not be suitable for *Pm*FREP. Some protein expression is detected in mammalian cells, but scaling up protein production would be cost prohibitive, especially with transient transfection. Appreciable yield was observed when *Pm*FREP was expressed in insect cells for both His_6_ *Pm*FREP and His_6_ *Pm*FREP CRD. Therefore, the insect cell expression is the system of choice for *Pm*FREP. XEEL signal peptide was used because of the observation that XEEL, a fibrinogen-related lectin of the intelectin family, is highly expressed in insect cells^12^. Cleavage of the signal peptide occurred at the expected site as confirmed by N-terminal protein sequencing. We reasoned that the placement of the His_6_ tag at the N-terminus is more suitable because the C-terminal carboxyl group of other fibrinogen-related lectins, such as H-ficolin^26^ or intelectins^12,13^, form a salt bridge with another amino acid residue. Placement of a C-terminal tag may disrupt this interaction and destabilize *Pm*FREP. In this case of human intelectin and XEEL, placement of the C-terminal tag drastically reduced the protein expression yield^12,13^. Utilization of the His_6_ tag is ubiquitous in biochemistry. The tag is placed at a protein terminus, which is not likely involved in interaction interfaces or any functional site^27^. Another advantage of the His_6_ tag is its function as an epitope tag. Thus, raising a specific antibody to *Pm*FREP is not required. Moreover, having a tag separated from the protein sequence allow better control over future immunoprecipitation experiments because antibody binding to the tag will be less likely to interfere with interaction of *Pm*FREP and other binding partners.

Because most lectins are oligomeric and the N-terminus of *Pm*FREP contains an odd number of 3 cysteines (C34, C50, and C58), we reason that *Pm*FREP might be able to form intermolecular disulfide bonds. SDS-PAGE analysis of His_6_ *Pm*FREP and His_6_ *Pm*FREP CRD under reducing and non-reducing conditions revealed that His_6_ *Pm*FREP is a disulfide-linked oligomer. The cysteines in the CRD are conserved among other fibrinogen-related lectins and are only involved in intramolecular disulfide bond formation^21,26^. This is consistent with the results that His_6_ *Pm*FREP CRD is monomeric both in reducing and non-reducing conditions. Because of this intermolecular disulfide bonds, it is unlikely that recombinant *Pm*FREP produced in *E. coli*, even if expressed, will be fully functional. Further examination of His_6_ *Pm*FREP quaternary structure by TEM revealed dumbbell-shaped molecules. Two-dimensional class averaging suggested that His_6_ *Pm*FREP is a dimer of pentamer with a flexible linker. The molecular weight of the dimer of pentamer, or decamer, would be around 345 kDa, which is consistent with the observation that His_6_ *Pm*FREP ran at more than 315 kDa in SDS-PAGE under non-reducing conditions. DLS also confirmed the particle size observed in TEM. Examination of His_6_ *Pm*FREP CRD by TEM revealed protein molecules about the size of the pentamer in the full length His_6_ *Pm*FREP. However, 2D class averaging did not yield sensible solutions, suggesting that His_6_ *Pm*FREP CRD might be able to oligomerize in solution, but the structure is rather inhomogeneous. Chemical cross-linking of His_6_ *Pm*FREP CRD in solution and DLS also indicate that the pentameric assembly of His_6_ *Pm*FREP CRD likely exists in solution. A similar molecular property is observed in XEEL, a fibrinogen-related lectin of the intelectin family^12^. XEEL is a dimer of trimer, and the CRD is capable of trimerization in solution even in the absence of the intermolecular disulfide bonds.

His_6_ *Pm*FREP recognized GlcNAc, as expected from structural homology to other fibrinogen-related lectins (Fig. 5). Most residues in the binding sites that interact directly with GlcNAc (R235, NH of C268, H269, Y285, and Y297) are conserved between *Pm*FREP and tachylectin 5A^21^. The residues recognizing the N-acetyl group (NH of C268, H269, Y285, and Y297) are conserved in human M-ficolin as well^28^. *Pm*FREP could also bind GalNAc, ManNAc, and Neu5Ac. The red blood cell agglutination experiments suggested that *Pm*FREP have the highest affinity towards ManNAc. However, the lack of experimental structural data, which we will continue to investigate, do not currently allow us to comments on specific interactions. It is also not clear whether ManNAc is a biologically relevant epitope since there is limited information on glycobiology of shrimp diseases. However, because *Pm*FREP was first identified as a peptidoglycan-binding lectin^23^, the GlcNAc-binding activity might still be biologically relevant.

The calcium ion binding residues (D247 and D249) are conserved between *Pm*FREP, tachylectin 5A, and M-ficolin, suggesting that *Pm*FREP possesses Ca^2+^ ion binding activity. However, our results showed that Ca^2+^ ion was not required for GlcNAc binding. Because the Ca^2+^ ion is not directly participating in ligand binding, it is possible that the Ca^2+^ ion may only have structural and stability role for *Pm*FREP. There are reports that other shrimp fibrinogen-related lectins require Ca^2+^ ion for ligand binding^29–33^. However, several ficolins are also capable of Ca^2+^-independent ligand binding^19,34–36^. The role of Ca^2+^ ion in *Pm*FREP ligand binding modulated by protein stability need to be further investigated. In contrast to the full length His_6_ *Pm*FREP, His_6_ *Pm*FREP CRD did not bind the GlcNAc affinity resin. The reduction in ligand binding affinity could be due to the lack of intermolecular disulfide bonds that may stabilize the structure. This observation is consistent with the TEM results which indicates that His_6_ *Pm*FREP CRD has relatively low structural homogeneity and may not be stable enough to bind GlcNAc with high affinity. In addition to potential reduction in structural stability, truncation of the full length protein to merely the CRD certainly reduced the multivalent binding capability of His_6_ *Pm*FREP. Multivalent binding event is well documented and is utilized ubiquitously in nature to increase apparent binding affinity, or avidity, especially in immune proteins and signaling events^37–39^. Therefore, reduction in binding avidity is expected for any lectin when the oligomeric state is reduced.

His_6_ *Pm*FREP clearly recognized *P. aeruginosa*. Binding is less strong towards *B. subtilis*, *S. aureus*, *E. coli*, and *V. parahaemolyticus*. Because His_6_ *Pm*FREP is a dimer of pentamer, the molecule should be able to engage two bacteria simultaneously and cause agglutination. Indeed, His_6_ *Pm*FREP agglutinated *P. aeruginosa* and this activity is inhibited by the presence of GlcNAc. Addition of EDTA to sequester Ca^2+^ ion did not inhibit agglutination. These results are consistent with the ligand binding and red blood cell agglutination experiments which suggested that Ca^2+^ is not required for ligand binding. In contrast to *P. aeruginosa*, His_6_ *Pm*FREP did not agglutinate *V. parahaemolyticus*, which is consistent with the weaker binding affinity of His_6_ *Pm*FREP towards *V. parahaemolyticus* in the ligand binding assay.

In conclusion, we have characterized the recombinant fibrinogen-like lectin *Pm*FREP from *P. monodon* expressed in insect cells. The protein is a dimer of pentamer with intermolecular disulfide bonds at the N-terminus. This dimer of pentamer molecular architecture is novel among fibrinogen-related lectins. *Pm*FREP binds GlcNAc like other fibrinogen-related lectins, such as tachylectin 5A and ficolins. In addition, binding to GalNAc, ManNAc, and Neu5Ac was observed. Ca^2+^ is not required for ligand binding, but may have structural roles as the calcium binding residues are conserved. The CRD itself is capable of oligomerization in solution, but cannot bind the GlcNAc ligand. *Pm*FREP recognizes and agglutinates *P. aeruginosa* in a Ca^2+^-independent manner, but cannot agglutinate the shrimp pathogen *V. parahaemolyticus* although weak binding is observed in the bacteria binding assay. The information obtained and His_6_ *Pm*FREP produced in the study will be useful for further biochemical investigation and the signaling pathway of innate immune lectins in shrimp that may help prevent and treat bacterial infectious disease in shrimp in the future.

## Methods

### Expression plasmids and protein expression

For expression in insect cells, the open reading frame for *Pm*FREP (GenBank accession number AIE45535) was amplified from *P. monodon* hemocyte cDNA by the primers 5′-GCGCGGATCCATGGCGCTCTTGCACAAGTTCATG-3′ and 5′-ATGCGGTACCTCATTAGAATGCCGGCCTTATCATCATTGTTG-3′, and cloned into the BamHI and KpnI sites of pFastBac1 (pFastBac1 *Pm*FREP). After sequencing, G184D substitution was noted in all the 3 clones sequenced and was thus assumed to be a natural variation. The plasmid for expression of *Pm*FREP His6 was made similarly, but with 5′-ATGCGGTACCTCATTAATGGTGATGGTGGTGATGGAATGCCGGCCTTATCATCATTGTTG-3′ as the reverse primer (pFastBac1 *Pm*FREP His_6_). To create the expression construct for His_6_ *Pm*FREP with *Xenopus laevis* embryonic epidermal lectin signal peptide (XEEL SP), pFastBac1 *Pm*FREP was used as a template to amplify with the primer 5′-CCAGCAGGGCACGCTGGTTCACATCACCACCATCACCACAGCGGTACAACAGAACGAACAGATACCGCGG-3′ and the same reverse primer used to make pFastBac1 *Pm*FREP. The PCR product was amplified again with the primers 5′-ATGCGGTACCATGTTGTCATATAGCCTGTTGCTTTTTGCACTTGCATTTCCAGCAGGGCACGCTGGTTCA-3′ and the same reverse primer. The PCR product was then cloned into the BamHI and KpnI sites of pFastBac1 (pFastBac1 XEEL SP His_6_ *Pm*FREP). To create the expression construct for the carbohydrate recognition domain of *Pm*FREP with the XEEL signal peptide (XEEL SP His_6_ *Pm*FREP CRD), pFastBac1 *Pm*FREP was used as a template to amplify with the primer 5′- CCAGCAGGGCACGCTGGTTCACATCACCACCATCACCACGGTTCACGGCCGAGGCACTGCCGCGACCTGC-3′ and the same reverse primer used to make pFastBac1 *Pm*FREP. The PCR product was re-amplified and cloned into pFastBac1 in the same manner as pFastBac1 XEEL SP His_6_ *Pm*FREP (pFastBac1 XEEL SP His_6_ *Pm*FREP CRD). Insect cell transfection, baculovirus production in Sf21, and protein production in *Trichoplusia ni* were carried out as described previously^12^.

The bacterial expression vectors were constructed using pFastBac1 *Pm*FREP His_6_ as a template for PCR. For the expression plasmid of *Pm*FREP His_6,_ the open reading frame was amplified using the primers 5′-TGGCCATGGGGACAACAGAACGAACAGATACC-3′ and 5′-TATACTCGAGGAATGCCGGCCTTATCATCATTG-3′, and then cloned into the NcoI and XhoI sites of pET28a (pET28a *Pm*FREP His_6_). For the expression plasmid of His_6_ *Pm*FREP, the open reading frame was amplified using the primers 5′-ATACCATGGGCCATCATCATCATCATCACGGGACAACAGAACGAACAGATACCG​-3′ and 5′- CTCGGATCCTCATTAGAATGCCGGCCTTATCATC -3′, and then cloned into the NcoI and BamHI sites of pET28a (pET28a His_6_ *Pm*FREP). For protein expression in *Escherichia coli*, the plasmids were each transformed into Tuner(DE3) and Rosetta(DE3). After growing at 37 °C until OD600 reached 0.6, protein expression was induced by 0, 0.5, and 1 mM IPTG for 6 h. Cells were collected by centrifugation and lysed by sonication. The soluble and insoluble fractions were analyzed by SDS-PAGE and western blot.

The mammalian expression plasmid for His_6_ *Pm*FREP with the XEEL signal peptide was constructed by PCR amplification of the XEEL SP His_6_ *Pm*FREP open reading frame from pFastBac1 XEEL SP His_6_ *Pm*FREP using the primers 5′-ATGCGGTACCATGTTGTCATATAGCCTGTTGCTTTTTGCACTTGCATTTCCAGCAGGGCACGCTGGTTCA-3′ and 5′-CTCGGATCCTCATTAATGGTGATGGTGGTGATGG-3′, and then cloned into the KpnI and BamHI sites of pcDNA4 myc His A (pcDNA4 myc His A XEEL SP His_6_ *Pm*FREP). For protein expression in mammalian cells, HEK293T was cultured in Dulbecco's Modified Eagle Medium (DMEM) supplemented with 10% fetal bovine serum and 100 U/mL of penicillin–streptomycin (Gibco). Prior to transfection, cells were plated at 10^6^ cells/well in a 6-well plate and incubated overnight. The transfection mixture contains 2 μg plasmid and 6 μg PEI (linear MW 25,000, Polysciences) in 200 μL of Opti-MEM (Gibco). After 30 min incubation, Opti-MEM was added to the total volume of 1 mL. The culture media was aspirated from the adherent cells and replaced with the transfection mixture. After 4 h, SFM4HKE293 (1.5 mL, HyClone) was added. Protein secretion was allowed to proceed for 48 h before the culture media was collected for analysis by western blot.

### Purification of His_6_*Pm*FREP and His_6_*Pm*FREP CRD

Insect culture media containing secreted protein were dialyzed against 20 mM Bis–Tris pH 6.5 and 150 mM NaCl to reduce media component precipitation and subsequently dialyzed against 20 mM HEPES pH 7.5, 150 mM NaCl, and 25 mM imidazole (loading buffer). The dialyzed insect culture media was then applied a Ni–NTA column equilibrated with the loading buffer. The column was washed with the loading buffer and the protein eluted with 20 mM HEPES pH 7.5, 150 mM NaCl, and 250 mM imidazole. Buffer was exchanged to 20 mM HEPES pH 7.5, 150 mM NaCl, and 10 mM CaCl_2_ by dialysis. The purity of the protein was examined by SDS-PAGE and the presence of the His_6_ tag verified by western blot. Protein concentrations were determined using spectrophotometry at 280 nm. For His_6_ *Pm*FREP (34.5 kDa) the extinction coefficient is 63,745 M^−1^ cm^−1^ or 1 absorbance unit = 1.849 mg/mL. For His_6_ *Pm*FREP CRD (26.6 kDa) the extinction coefficient is 63,620 M^−1^ cm^−1^ or 1 absorbance unit = 2.396 mg/mL. N-terminal sequencing of the purified proteins was performed with ABI 494 Protein Sequencer (Tufts University Core Facility, Tufts Medical School). Dynamic light scattering data were collected on Malvern Zetasizer Nano ZS using microcuvettes (40 µL).

### Negative stain and transmission electron microscopy

Samples were diluted in 5 mM HEPES pH 7.5, 1 mM CaCl_2_ to concentration of 5 µg/mL. Negative stain was carried out as previously described^40^. TEM data acquisition was performed on FEI Tecnai T12 electron microscope operating at 120 kV equipped with 4 k x 4 k CCD camera (Gatan Ultrascan). Images were taken at magnifications of 110,000 (0.7 Å/pixel, defocus − 0.5 μm). Images format conversion was performed with EMAN2^41^. Image processing and 2D class averaging were performed with cisTEM^42^.

### His_6_*Pm*FREP CRD crosslinking

Bis(sulfosuccinimidyl)suberate (BS3, Pierce) was dissolved in water and added to 90 µL solutions of His_6_ *Pm*FREP CRD (80 µg/mL in 20 mM HEPES pH 7.5, 150 mM NaCl, and 10 mM CaCl_2_) to achieve the final concentrations of 0, 0.05, 0.1, 0.25, 0.5, 1, 2.5, and 5 mM with the total volume of 100 µL. The reaction mixture was incubated at room temperature for 30 min and the quenched by addition of 1 M Tris pH 7.5 to the final concentration of 20 mM. The product was then analyzed by western blot.

### Carbohydrate binding assay

Affinity resins containing different carbohydrate ligands were prepared as previously described^43^. Purified His_6_ *Pm*FREP or His_6_ *Pm*FREP CRD in 20 mM HEPES pH 7.5, 150 mM NaCl, and 10 mM CaCl_2_ (binding buffer). The protein solution (20 µg/mL, 100 µL) was then applied to 100 µL of affinity resin in Bio-Rad Micro Bio-Spin column that had previously been washed with water and pre-equilibrated with the binding buffer. After centrifugation, the resin was washed with 3 × 250 µL of the binding buffer. The bound protein was then eluted with 2 × 100 µL of the elution buffer and analyzed by western blot with anti-His_6_ antibody as the primary antibody. For competitive elution with monosaccharide, the elution buffer is 20 mM HEPES pH 7.5, 150 mM NaCl, 10 mM CaCl_2_, and 100 mM monosaccharide. For EDTA elution, the elution buffer is 20 mM HEPES pH 7.5, 150 mM NaCl, and 10 mM EDTA. The eluted samples were precipitated in cold acetone (4 volume) at − 20 °C for 2 h. The solution was centrifuged at 14,000×*g* at 4 °C for 30 min. The supernatant was removed and the pellet dried at room temperature. The precipitate was dissolves in SDS-PAGE sample buffer and subjected to Western blot using anti-His_6_ antibody.

### Red blood cell agglutination assay

Human red blood cells were purchased from Thai Red Cross and washed with the binding buffer before use. His_6_ *Pm*FREP was incubated with a suspension of 3% (v/v) human red blood cells (A, B, and O) in 72-well Terasaki plates. In the inhibition experiments, various sugars were incubated with 0.5 μM His_6_ *Pm*FREP and 3% (v/v) human red blood cells was then added, agglutination activity was observed as diffused cells that do not settle to the bottom of the well compared to untreated cells.

### Bacteria binding and agglutination assay

All bacteria were cultured in nutrient broth, except *V. parahaemolyticus* that was cultured in nutrient broth with 3% NaCl. Bacteria (50 mg) were pelleted and washed with 250 µL of the binding buffer. The pellets were then suspended in His_6_ *Pm*FREP (20 µg/mL, 250 µL) then incubated on ice for 10 min. The pellets were washed with 3 × 250 µL of the binding buffer. The protein was then eluted from each pellet by 100 µL of 20 mM HEPES pH 7.5, 150 mM NaCl, 10 mM CaCl_2_, and 100 mM monosaccharide. The eluted protein was analyzed by western blot with anti-His_6_ antibody as the primary antibody.

For the agglutination assay, the bacteria were grown, washed, and resuspended in the binding buffer as mentioned above. The bacteria solution (50 µL) was mixed with His_6_ *Pm*FREP (100 µg/mL or 3 µM, 10 µL). The total volume was then adjusted to 70 µL with the binding buffer with or without the addition of GlcNAc or EDTA to the final concentration of 10 or 25 mM, respectively. A 20 µL sample of each mixture was then photographed under an Olympus CX31 light microscope equipped with a Canon EOS 650D digital camera.

## Supplementary Information

Supplementary Information
