# Supplementary material for: Biochemical and structural characterization of a recombinant fibrinogen-related lectin from Penaeus monodon
Source: Sci Rep. 2021 Feb 3;11:2934. doi: 10.1038/s41598-021-82301-5 (PMC7858579; doi:10.1038/s41598-021-82301-5)
Supplement: Supplementary file 1 — Supplementary Information [file 41598_2021_82301_MOESM1_ESM.docx]

**Supplementary Materials**

**Biochemical and structural characterization of a recombinant fibrinogen-related lectin from *Penaeus monodon***

Nongnuch Singrang^1^, Sirasit Laophetsakunchai^1^, Bich Ngoc Tran^2^, Paul T. Matsudaira^2^, Anchalee Tassanakajon^1^, Kittikhun Wangkanont^1,*^

^1^ Center of Excellence for Molecular Biology and Genomics of Shrimp, and Molecular Crop Research Unit, Department of Biochemistry, Faculty of Science, Chulalongkorn University, Bangkok, Thailand

^2^ Centre for BioImaging Sciences, Department of Biological Sciences, Faculty of Science, National University of Singapore

*To whom correspondence should be addressed: kittikhun.w@chula.ac.th


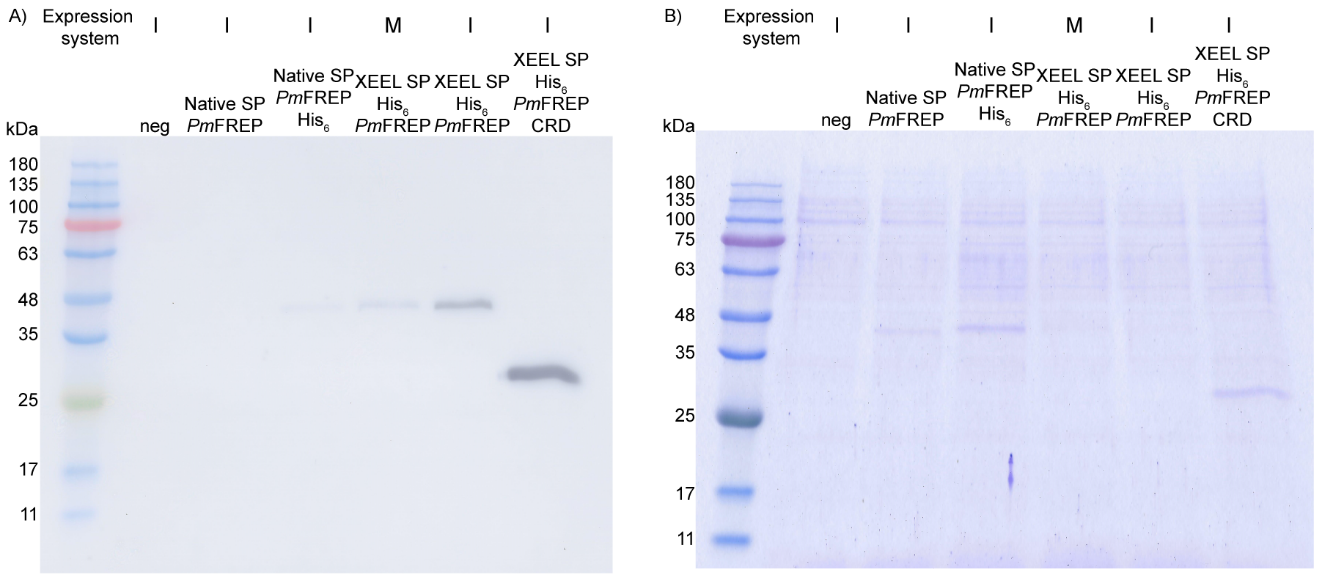


**Figure S1**: Expression screen of various *Pm*FREP constructs by A) Western blot probed with an anti-His_6_ antibody and B) SDS-PAGE with Coomassie Blue stain. I = insect, M = mammalian.


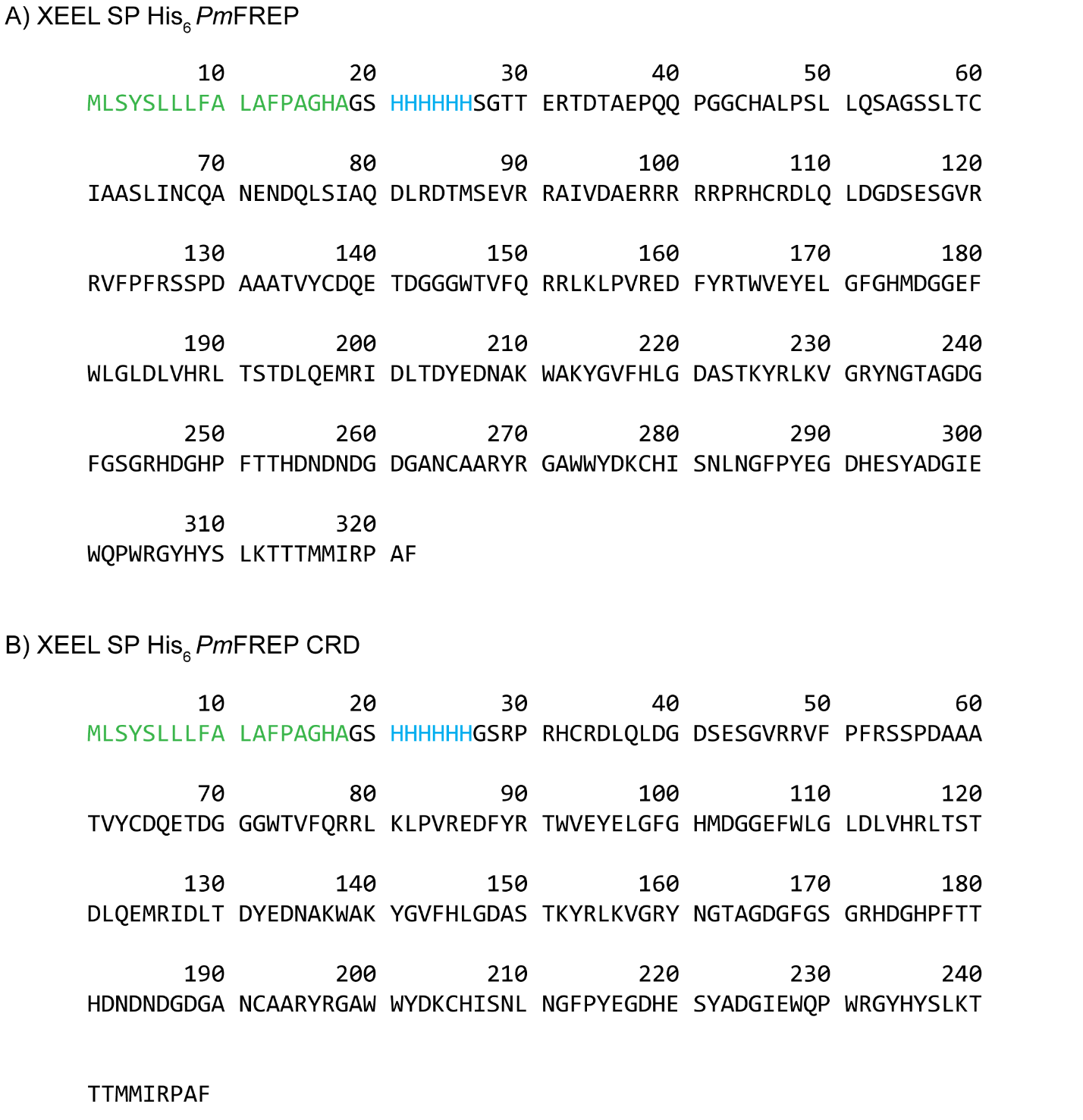


**Figure S2**: Sequences of the constructs used in this study A) XEEL SP His_6_ *Pm*FREP and B) XEEL SP His_6_ *Pm*FREP CRD. XEEL SP is shown in greed and the His_6_ tag is shown in blue.


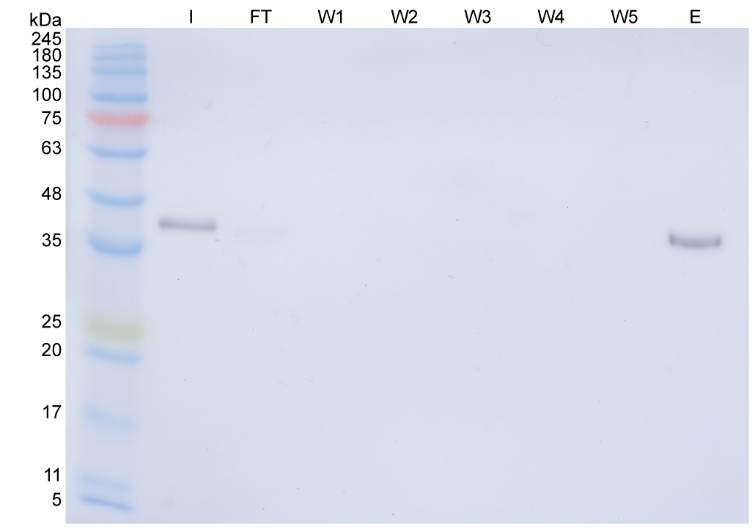


**Figure S3**: Purification of His_6_ *Pm*FREP with Ni-NTA agarose monitored by western blot probed with anti-His_6_ antibody. I = input, FT = flow through, W = wash, and E = elute

**
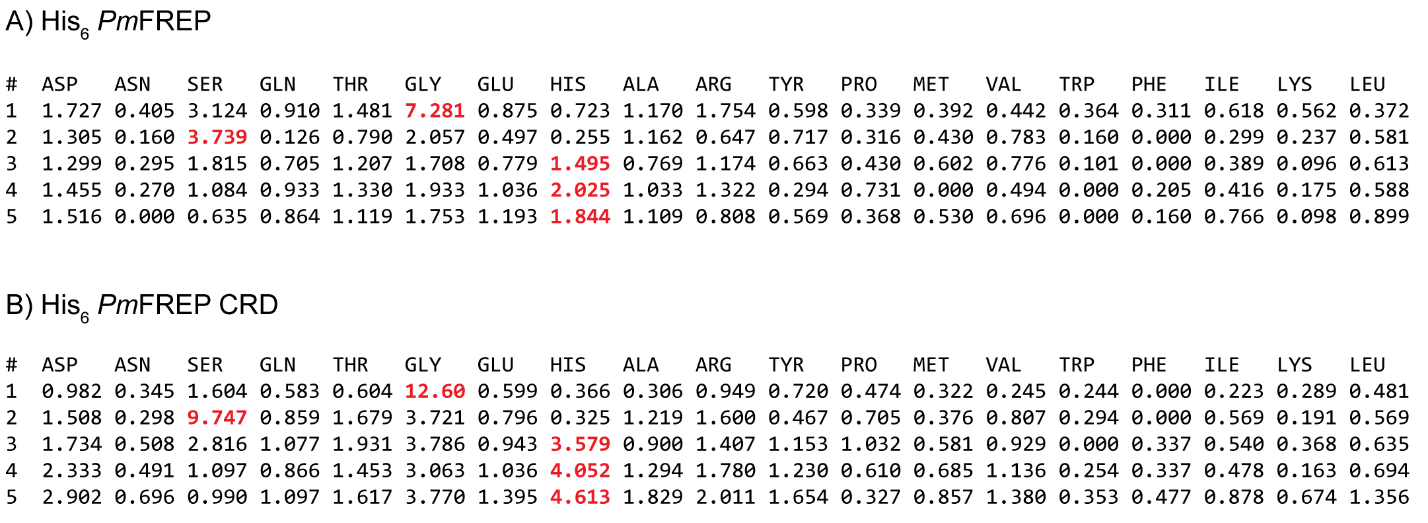
**

**Figure S4**: N terminal sequencing of purified A) His_6_ *Pm*FREP and B) His_6_ *Pm*FREP CRD. The numbers represent pmol amount of phenylthiohydantoin derivatives of amino acids from each cleavage cycle. Red values indicate the likely amino acid that was cleaved in each cycle.


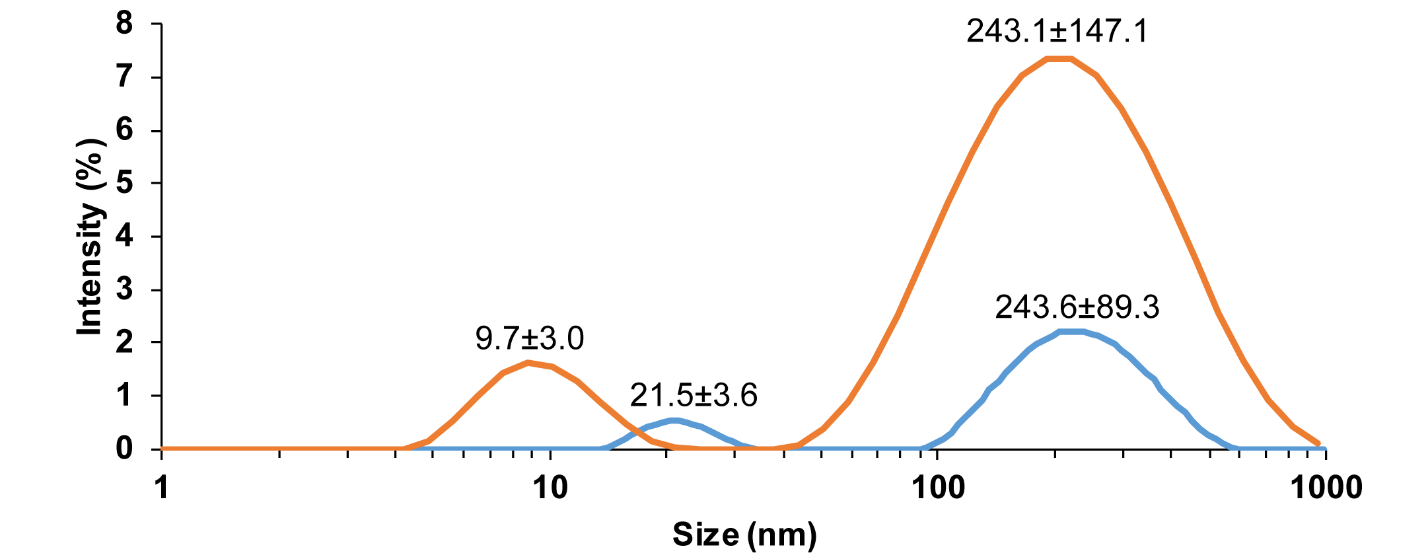


**Figure S5:** Size distribution of His_6_ *Pm*FREP (22 µg/mL, blue) and His_6_ *Pm*FREP CRD (125 µg/mL, orange) obtained by dynamic light scattering experiments. The numbers on the peaks are particle size in nanometer (nm). The particle size around 243 nm is present in all of our experiments and is too large to be protein, thus likely dust particles.
